# Correction: Knockout of a single aquaporin, OsPIP2;4, decreases root water permeability in rice

**DOI:** 10.1007/s10265-026-01713-4

**Published:** 2026-05-23

**Authors:** Aya Onishi, Tomoaki Horie, Ryo Ishitsuka, Shizuka Sasano, Rie Horie, Yunosuke Mito, Shigeko Utsugi, Junko Ishikawa, Majid Mahdieh, Maki Katsuhara

**Affiliations:** 1 Institute of Plant Science and Resources, Okayama University, 2-20-1 Chuo, Kurashiki, Okayama 710-0046 Japan; 2 Division of Applied Biology, Faculty of Textile Science and Technology, Shinshu University, 3-15-1 Tokida, Ueda, Nagano 386-8567 Japan; 3 Institute of Crop Science, NARO, 2-1-2 Kannondai, Tsukuba, Ibaraki 305- 8518 Japan; 4 Present Address: NARO, 3-1-1 Kannondai, Tsukuba, Ibaraki 305-8517 Japan; 5 Department of Biology, Faculty of Science, Arak University, Arak, 381568-8349 Iran

**Correction to: Journal of Plant Research (2026) 139:255–265, **10.1007/s10265-025-01691-z.

In the original version of this article, there were few errors in the author names and affiliations which are corrected as in this correction.

1. The given and family names of Aya Onishi, Tomoaki Horie were incorrectly structured. The name was displayed correctly in all versions at the time of publication.

2. Tomoaki Horie is affiliated with ‘Division of Applied Biology, Faculty of Textile Science and Technology, Shinshu University, 3-15-1 Tokida, Ueda, Nagano 386-8567, Japan’.

3. Majid Mahdieh is affiliated with ‘Institute of Plant Science and Resources, Okayama University, 2–20-1 Chuo, Kurashiki, Okayama 710-0046, Japan’ and ‘Department of Biology, Faculty of Science, Arak University, 381,568-8349, Arak, Iran’.

The original article has been corrected.

